# Comparing Self-Monitoring Strategies for Weight Loss in a Smartphone App: Randomized Controlled Trial

**DOI:** 10.2196/12209

**Published:** 2019-02-28

**Authors:** Michele L Patel, Christina M Hopkins, Taylor L Brooks, Gary G Bennett

**Affiliations:** 1 Department of Psychology and Neuroscience Duke University Durham, NC United States; 2 Duke Digital Health Science Center Duke Global Health Institute Durham, NC United States; 3 Stanford Prevention Research Center Stanford University School of Medicine Palo Alto, CA United States

**Keywords:** weight loss, obesity, self-monitoring, technology, mobile app, mobile health, caloric restriction, treatment adherence and compliance, randomized controlled trial

## Abstract

**Background:**

Self-monitoring of dietary intake is a valuable component of behavioral weight loss treatment; however, it declines quickly, thereby resulting in suboptimal treatment outcomes.

**Objective:**

This study aimed to examine a novel behavioral weight loss intervention that aims to attenuate the decline in dietary self-monitoring engagement.

**Methods:**

GoalTracker was an automated randomized controlled trial. Participants were adults with overweight or obesity (n=105; aged 21-65 years; body mass index, BMI, 25-45 kg/m^2^) and were randomized to a 12-week stand-alone weight loss intervention using the MyFitnessPal smartphone app for daily self-monitoring of either (1) both weight and diet, with weekly lessons, action plans, and feedback (Simultaneous); (2) weight through week 4, then added diet, with the same behavioral components (Sequential); or (3) only diet (App-Only). All groups received a goal to lose 5% of initial weight by 12 weeks, a tailored calorie goal, and automated in-app reminders. Participants were recruited via online and offline methods. Weight was collected in-person at baseline, 1 month, and 3 months using calibrated scales and via self-report at 6 months. We retrieved objective self-monitoring engagement data from MyFitnessPal using an application programming interface. Engagement was defined as the number of days per week in which tracking occurred, with diet entries counted if ≥800 kcal per day. Other assessment data were collected in-person via online self-report questionnaires.

**Results:**

At baseline, participants (84/100 female) had a mean age (SD) of 42.7 (11.7) years and a BMI of 31.9 (SD 4.5) kg/m^2^. One-third (33/100) were from racial or ethnic minority groups. During the trial, 5 participants became ineligible. Of the remaining 100 participants, 84% (84/100) and 76% (76/100) completed the 1-month and 3-month visits, respectively. In intent-to-treat analyses, there was no difference in weight change at 3 months between the Sequential arm (mean −2.7 kg, 95% CI −3.9 to −1.5) and either the App-Only arm (−2.4 kg, −3.7 to −1.2; *P*=.78) or the Simultaneous arm (−2.8 kg, −4.0 to −1.5; *P*=.72). The median number of days of self-monitoring diet per week was 1.9 (interquartile range [IQR] 0.3-5.5) in Sequential (once began), 5.3 (IQR 1.8-6.7) in Simultaneous, and 2.9 (IQR 1.2-5.2) in App-Only. Weight was tracked 4.8 (IQR 1.9-6.3) days per week in Sequential and 5.1 (IQR 1.8-6.3) days per week in Simultaneous. Engagement in neither diet nor weight tracking differed between arms.

**Conclusions:**

Regardless of the order in which diet is tracked, using tailored goals and a commercial mobile app can produce clinically significant weight loss. Stand-alone digital health treatments may be a viable option for those looking for a lower intensity approach.

**Trial Registration:**

ClinicalTrials.gov NCT03254953; https://clinicaltrials.gov/ct2/show/NCT03254953 (Archived by WebCite at http://www.webcitation.org/72PyQrFjn).

## Introduction

### Background

Self-monitoring of dietary intake is a cornerstone of behavioral weight loss treatment [1], and past research has demonstrated that the frequency of self-monitoring is positively associated with weight loss [2]. Despite its utility, dietary self-monitoring typically declines over the course of treatment [2,3]. Novel strategies are needed to improve dietary self-monitoring engagement [4]. One strategy involves enriching self-monitoring with other theoretically and empirically supported behavior change techniques such as tailored goals and feedback, action plans, and skills training [5,6].

A second strategy includes building mastery, self-efficacy, and self-regulation—key constructs of behavior change in Carver’s Control Theory [7] and Bandura’s Social Cognitive Theory [8]— *before* asking participants to engage in dietary self-monitoring. Fostering self-regulatory skills may provide an opportunity for mastery and, in turn, strengthen self-efficacy [9], which has been linked to greater weight reduction [10]. Behavioral weight loss interventions typically involve self-monitoring multiple behaviors or outcomes simultaneously during treatment [2,11,12], which can serve as an efficient strategy for producing behavior change but may detrimentally impede performance on each item or result in greater treatment dropout [13,14]. Failing to develop mastery of a behavior can weaken self-efficacy [15], leading to worse treatment outcomes. In addition, when people begin to self-monitor a behavior less frequently, it is likely that they will also self-monitor a corresponding behavior less frequently, as demonstrated in an analysis of self-monitoring patterns [16].

We propose a novel solution that aims to attenuate the decline in engagement by employing a *sequential* [17] self-monitoring approach, wherein individuals track only body weight for a period of time and then begin to track diet. Tracking body weight was chosen as it requires minimal effort, provides an opportunity for habit formation (eg, track every morning upon waking), and is efficacious for weight loss [18]. We focused on only self-monitoring of body weight during the first month based on prior research demonstrating that enhanced engagement in the first month of treatment may have long-lasting repercussions [19]. Sequential approaches are based on the premise that mastery is more easily obtained if only 1 new behavior is targeted at a time [20,21]. Recent research finds that both sequential and simultaneous approaches are effective for behavior change [21] and that focusing on a single component instead of a multicomponent intervention produces comparable weight loss [22], suggesting that a simpler approach is sufficient to produce behavior change.

We tested this sequential strategy using a remotely delivered intervention that utilizes a popular commercially available mobile app—MyFitnessPal. Utilizing technology for self-monitoring dietary intake has been shown to produce greater adherence and less-pronounced declines in engagement than traditional paper-based tracking methods [23,24]. As demonstrated in recent reviews [25-27], smartphone apps can produce significant weight loss, although most existing trials that use commercial apps lack fully powered designs or are pilot studies. Interventions without counseling that utilize commercial technology for dietary self-monitoring have produced clinically meaningful weight losses between 2.5 kg and 5.5 kg at 3 months and beyond in recent studies [28-32].

### Objectives

In the current randomized trial, we test a weight loss intervention among adults with overweight or obesity that targets the aforementioned strategies of including empirically supported behavior change techniques, promoting mastery and self-efficacy through self-monitoring of body weight before diet, and utilizing a free, commercially available app (MyFitnessPal). We hypothesize that a sequential approach will produce greater weight loss and self-monitoring engagement at 3 months compared with a traditional simultaneous approach and to an “off-the-shelf” app.

## Methods

### Study Design Overview

GoalTracker was a 3-arm randomized controlled trial comparing 3 stand-alone weight loss interventions: (1) a *Simultaneous* self-monitoring arm in which participants simultaneously tracked body weight and dietary intake each day and received additional empirically supported behavior change techniques (see Table 1) via email for the entirety of the intervention, (2) a *Sequential* arm, consisting of identical intervention components but allowing for mastery of 1 skill (ie, self-monitoring of body weight) before beginning self-monitoring of diet, and (3) an *App-Only* arm that tracked only diet with no additional behavior change components. Study evaluation visits were held at baseline, 1 month, and 3 months. Self-reported weight was collected at 6 months. All study procedures were approved by the Duke University Institutional Review Board (protocol #: D0822; date of approval: 10/14/16).

**Table 1:** Differences in intervention components between treatment arms.

Intervention component	Sim^a^	Seq^b^	App-Only arm	Theoretical construct	Behavior change technique [5]
Weight loss goal: 0.5-2.0 lb/week (tailored) and 5% by 12 weeks	✓^c^	✓	✓	Self-regulation	Goal setting (outcome)
Calorie goal: tailored based on individual factors and rate of weight loss; minimum 1200 kcal for women, 1500 kcal for men	✓	✓ (delayed)	✓	Self-regulation	Goal setting (behavior)
Self-monitoring of body weight: daily via the app	✓	✓	—^d^	Self-regulation	Prompt self-monitoring of behavioral outcome
Self-monitoring of dietary intake: daily via the app	✓	✓ (delayed)	✓	Self-regulation	Prompt self-monitoring of behavior
Facilitate mastery experience by first tracking weight then tracking diet	—	✓	—	Self-efficacy; self-regulation; mastery	Set graded tasks
In-app real-time feedback	✓	✓	✓	Self-regulation; self-efficacy	Provide feedback on performance
Out-of-app summary feedback via weekly email (tailored)	✓	✓	—	Self-regulation; self-efficacy	Provide feedback on performance; prompt review of outcome goals; prompt review of behavioral goals
Skills training via weekly email with structured behavioral lesson and tips on how to use features of the app	✓	✓	—	Outcome expectancies; self-efficacy	Provide information on consequences of behavior in general; prompt generalization of a target behavior; provide information on when and where to perform the behavior; provide instruction on how to perform the behavior; environmental restructuring; plan social support/social change; relapse prevention/coping planning
Action plans via weekly email	✓	✓	—	Self-regulation; self-efficacy	Action planning; motivational interviewing [33]; barrier identification/problem solving [34]; prompt practice; plan social support/social change
Reminder of goals	✓	✓	—	Self-regulation	Prompt review of outcome goals; prompt review of behavioral goals
In-app automated reminders to track diet and/or weight sent daily (App-Only received reminders to track diet, Simultaneous received both diet and weight tracking reminders, and Sequential received weight tracking for all 12 weeks and diet tracking reminders starting in week 5)	✓	✓	✓	Self-regulation	Teach to use prompts/cues

^a^Sim: Simultaneous self-monitoring intervention arm.

^b^Seq: Sequential self-monitoring intervention arm.

^c^The component is present.

^d^The component is not present.

### Participants

Inclusion criteria comprised men and women aged 21 to 65 years with a body mass index (BMI) between 25.0 and 45.0 kg/m^2^ who were interested in losing weight through dietary change. We required participants to have an iPhone or Android smartphone, email address, access to a bathroom scale, and written English fluency. Participants needed to be willing to download the mobile app on their phone and not track diet or body weight using any other modality (eg, other health or weight tracking apps, websites, and paper diaries) for the duration of the intervention. We excluded participants if they were enrolled in another weight loss intervention, had used MyFitnessPal to track diet in the past 6 months, had lost ≥10 lb or used a weight loss medication in the past 6 months, had previous or planned bariatric surgery, or if weight loss would be contraindicated (eg, pregnancy or <12 months postpartum or in need of medical or psychiatric intervention such as for cancer diagnosis, eating disorder, uncontrolled hypertension, diabetes mellitus, cardiovascular event, or congestive heart failure). A total of 2 criteria were amended during the trial recruitment to promote generalizability of findings: the BMI criteria were expanded to include participants in the 40.0 to 45.0 kg/m^2^ range, and the weight change criteria were adjusted to no longer exclude individuals who gained more than 10 lb in the past 6 months. The institutional review board approved both amendments.

### Recruitment

Recruitment occurred between April and September 2017 in central North Carolina via a university-affiliated research website and listservs, social media postings (Twitter and Facebook), ClinicalTrials.gov registry, and community advertisements (Craigslist, Nextdoor, and paper flyers). Advertisements provided a description of the study and eligibility criteria. Participants were enrolled on a rolling basis until we met our intended sample size.

### Procedure

We directed interested individuals to a study website with descriptive information and a screening questionnaire that assessed all eligibility criteria, including participants’ height and weight. Study personnel contacted eligible candidates within 3 business days to schedule an in-person baseline visit. During the baseline visit, trained study staff obtained written informed consent, confirmed eligibility, collected anthropometric measurements, and assisted participants in installing and navigating the MyFitnessPal mobile app; participants then completed an online survey.

Using simple random assignment, participants were then randomized by study staff to 1 of 3 treatment arms using Excel’s random number generator to allocate participants equally (1:1:1) across conditions. Randomization was revealed to participants by study personnel; as such, study staff were not blinded to treatment allocation but were blinded to the allocation sequence. Participants then reviewed materials describing their treatment condition and goals (see Intervention Design section below) in writing and with study staff to reduce contamination.

In-person follow-up visits occurred at 1 month and 3 months. At 1 month, study staff provided participants with information on their goals for the remainder of the intervention (see Intervention Design section below for details). We compensated participants with Amazon electronic gift cards (US $12 at baseline, US $6 for each follow-up visit, and US $5 bonus for completing dietary measures). Questionnaires were administered in English via a desktop computer. There was no contact with participants from months 3 to 6, and participants were not asked to self-monitor in MyFitnessPal during this time (though they could still do so if desired). At 6 months, study staff contacted participants via email and text message to collect self-reported body weight. Data collection ended in March 2018.

### Intervention Design

Participants were randomized to 1 of 3 conditions: (1) Simultaneous, (2) Sequential, or (3) App-Only, as outlined below and in Table 1. The CALO-RE taxonomy is used to describe behavior change techniques [5]. The intervention period lasted 12 weeks.

#### Common Components

All treatment arms self-monitored dietary intake using MyFitnessPal, a free commercial app that allows users to log food and beverages and provides nutritional information from a database with over 6 million foods [35]. This app has high acceptability [36]. In-app feedback in both graphical and text format provides users with real-time progress updates. When setting up participants’ MyFitnessPal accounts, study staff entered an end goal weight that corresponded with losing 5% of their initial body weight by 12 weeks. On the basis of this goal and the participant’s current weight, a weekly weight loss goal between 0.5 and 2.0 pounds was calculated. Along with the Mifflin-St. Jeor equation that factors in basal metabolic rate [37], this value was used to determine a tailored daily calorie goal, with a minimum caloric goal of 1200 kcal/day for women and 1500 kcal/day for men. During the baseline visit, in-app push-reminders were programmed to be sent each day if tracking had not occurred by a prespecified time in the evening. No structured dietary advice (eg, follow a low carbohydrate diet) was given to participants. Of note, study staff also created a Fitbit account for each person via the platform’s website and linked this account with MyFitnessPal. Participants were not given a Fitbit device and they were never asked to use this Fitbit account; its sole purpose was for accessing MyFitnessPal’s data using Fitbit’s application programming interface (API). In the App-Only arm, MyFitnessPal served as an “off-the-shelf,” self-guided approach that the general US population can already access for free in the commercial marketplace.

#### Both Simultaneous and Sequential Arms

In addition to the common intervention components, participants in the Simultaneous and Sequential arms were asked to self-weigh and enter their body weight in the app each day. Each week, study staff sent participants an email with tailored feedback that was automatically generated using Microsoft Word’s Mail Merge feature. This feedback email described the participants’ overall weight loss progress and their progress on each goal in the past week, including track weight daily, meet weekly weight loss goal, track diet daily, and meet daily calorie goal (the latter 2 goals not given to Sequential participants until week 5). Feedback on weight outcomes was provided as long as 1 weight was recorded in the past week. Individuals who did not track their weight in the past week received a message stating “Make sure to enter your weight in MyFitnessPal so that we can give you helpful insights!” Feedback pertaining to the calorie goal included only days with complete food diaries (≥800 kcal) [38]. This calorie feedback was not given to the Sequential arm until week 5.

Each week, participants were also sent skills training materials via email on a different day, including a researcher-designed tip on using different features of the app (eg, using the barcode scanner) accompanied by step-by-step screenshots of the app, a lesson on nutrition or behavior change (eg, reducing sugary foods and managing food intake on vacations; see Multimedia Appendix 1 for an example) adapted from gold-standard weight loss curriculum [39,40], and a brief online action plan to reinforce the weekly lesson. Accessed via a link to a Qualtrics survey, action plans incorporated motivational interviewing and problem-solving strategies [33,34] and included the following types of components: identifying current behaviors and beliefs; evaluating confidence and reasons for change; thinking about the *when*, *where*, and *what* of each action; brainstorming potential barriers that may arise and crafting solutions; identifying a support person; and reviewing past action plans (see Tables 2 and 3 for lesson and tip topics).

**Table 2:** Topics of structured lessons.

Week	Lesson topic
1	Overview of the program (losing 5% weight, self-monitoring); calorie balance^a^
2	Red zone foods; green zone foods
3	Reading food labels^a^
4	Reducing sugar
5	Portion control
6	Preparing meals at home; shopping tips
7	Eating out
8	Social support
9	Environmental cues; vacations and holidays
10	Emotional eating
11	Slippery slope; weight loss maintenance; relapse prevention

^a^In these 2 lessons for the Sequential treatment arm, there was no discussion of tracking diet or adhering to a calorie goal. Otherwise, all lesson content was identical between the arms.

**Table 3:** Tips for using the MyFitnessPal app.

Week	Tips for Simultaneous arm	Tips for Sequential arm
Sent after baseline visit	A: How to track body weight; B: How to view weight progress; C: How to track a food item; D: How to view your calorie goal and the foods you have tracked	A: How to track body weight; B: How to view weight progress
1	How to use the barcode scanner	How to delete a weight entry
2	How to use multiadd to speed up food tracking	How to add progress photos
3	How to view nutrition progress	How to change reminders to track (weight)
4	A: How to delete a weight entry; B: How to add progress photos	How to recruit a friend to use MyFitnessPal
Sent after 1MV^a^	—^b^	A: How to track a food item; B: How to view your calorie goal and the foods you have tracked
5	How to track food from a restaurant	How to use the barcode scanner
6	A: How to create a meal; B: How to log a meal	How to use multiadd to speed up food tracking
7	A: How to add a recipe; B: How to log a recipe	How to view nutrition progress
8	How to use the “Complete Diary” feature	How to track food from a restaurant
9	How to change reminders to track (weight and food)	A: How to create a meal; B: How to log a meal
10	How to customize meal names	A: How to add a recipe; B: How to log a recipe
11	How to recruit a friend to use MyFitnessPal	A: How to use the “Complete Diary” feature; B: How to customize meal names

^a^1MV: 1-month visit.

^b^Data are not applicable (no tips were provided to the Simultaneous arm directly after the 1-month visit).

#### Sequential Arm’s Self-Monitoring and Feedback

Individuals in the Sequential arm received the same intervention components as the Simultaneous arm, but they did not begin self-monitoring dietary intake until week 5 of the intervention. They did not receive a calorie goal until their 1-month evaluation visit, nor were their in-app reminders for tracking diet set up before this time point. The Sequential arm’s weekly feedback emails did not mention diet tracking or the calorie goal until they began tracking diet. In addition, their weekly app usage tips did not describe diet-tracking tips until after the first month (see Table 3). Like the Simultaneous arm, they were still encouraged to make healthy dietary changes during the first month as suggested in the weekly lessons and action plans, but these lessons did not mention tracking diet or adhering to a calorie goal.

### Outcome Measures

#### Primary Outcome: Change in Weight

The primary outcome was weight change at 3 months. We measured body weight using a calibrated electronic scale (SECA 876) at baseline, 1 month, and 3 months in light clothing with shoes removed. Height was measured to the nearest 0.1 cm using a calibrated, wall-mounted stadiometer (SECA 222). Baseline height was used for calculation of BMI at all time points. We collected self-reported body weight at 6 months and asked participants to send a photo with their feet on the scale displaying the value in either kg or lb. We assessed the proportion of participants at 3 months who achieved weight loss of ≥3% and ≥5% from baseline.

#### Self-Monitoring Engagement Data

We used a software engine developed at Duke—Prompt—to collect participants’ objective MyFitnessPal self-monitoring data; Prompt retrieved these data using the API of Fitbit, which was linked to each participant’s MyFitnessPal account. Primary outcomes for self-monitoring engagement span from day 1 (the day after participants’ baseline visit) to day 83 and were categorized into the first 4 weeks in the intervention (days 1-28), the final 2 months (days 29-83), and the entire 83-day intervention period. Exploratory analyses examined engagement data after the intervention ended up to 6 months (day 183) post randomization.

For all self-monitoring data, we only counted days with complete diet entries (ie, recording ≥800 kcal/day [38]). We examined the median number of days per week that participants self-monitored weight and diet, as well as the percentage of days that entries were recorded (ie, number of days with entries recorded divided by number of days instructed to record an entry, multiplied by 100).

#### Engagement in Action Plans

Percentage of action plans completed was examined through objective Qualtrics survey data: each action plan was coded as “completed” or “not completed.” The completion status of each action plan was combined to generate a summary score with a possible range of 0% to 100% (indicative of 11 out of 11 action plans completed).

#### Engagement in Feedback Email

In the 3-month survey, we assessed participants’ self-reported frequency of reading their weekly feedback email, with the question “How frequently did you read your weekly Progress Reports (sent via email), on average?” and 5 response options (*Several times per week*, *One time per week*, *Less than 1 time per week*, *Less than 1 time per month*, or *Never*).

#### Sociodemographic and Clinical Characteristics

At baseline, we collected data on participant demographics, socioeconomic status, and type of smartphone. To assess past MyFitnessPal use, we asked the Pew Research Center’s question: “What kind of health apps do you currently have on your phone?” [41]; if the “Diet, Food, Calorie Counter” response option was selected, then the open-ended question “What are the names of the diet, food, or calorie-counting apps that you used on your phone?” was asked.

We also assessed whether participants had ever been told by a doctor or other health professional that they had prediabetes or hypertension. Self-monitoring of weight and self-monitoring of diet in the month before baseline were each measured with a 7-point scale ranging from *several times per day* to *never* [42].

### Statistical Analysis

Sample size was calculated based on power to detect a 3.5 kg difference in weight change at 3 months between the Sequential arm and the App-Only arm (our primary comparison) using 3-month results from previous remotely delivered weight loss interventions for our Sequential arm [43] (results in kilograms were provided upon request by the author) and our App-Only arm [44]. Our power analysis (G*Power 3.1.9.2.) determined that 31 participants per group were needed to achieve 80% power for a 2-sided test with an alpha level of .05. To account for attrition of 10% and to obtain equal-size groups, we aimed to recruit 105 participants (35 per group). In exploratory analyses, we compared weight change between the Sequential arm and the Simultaneous arm, although we were not adequately powered to detect a significant effect.

For the baseline characteristics, we computed descriptive statistics stratified by treatment arm. To determine whether baseline characteristics differed by retention status, we used the Pearson chi-square test for categorical variables, analysis of variance for continuous variables, and Fisher exact tests with small cell counts. All analyses were 2-tailed. Participants who became ineligible during the study period up to 3 months were excluded from the analyses. Investigators remained blinded to outcomes until the completion of the 6-month trial.

We used intent-to-treat analyses to test our primary aim using linear mixed modeling with an unstructured covariance matrix and restricted maximum likelihood estimates to examine changes in weight over time by treatment arm. We did not control for any additional variables, and we assumed missing at random and used SAS 9.4 PROC MIXED (SAS Institute) for these analyses. For 6-month weight values sent via photo, we subtracted 0.172 kg (0.4 lb) to account for participants holding a device on the scale to take the photo. To account for the 6-month self-reported weight data without photos, we used a regression model to adjust for age, gender, and race/ethnicity [45]. Participants who sent a photo of their 6-month weight did not differ on any measured sociodemographic characteristics from those who did not send a photo (data not shown). We used chi-square tests to assess proportion of participants achieving ≥3% and ≥5% weight loss; we assumed noncompleters did not achieve this clinical threshold.

Given non-normally distributed intervention engagement data, we reported medians and interquartile ranges (IQR). To examine differences between treatment arms, we used Wilcoxon Mann-Whitney *U* tests (if 2 arms) and the Kruskal-Wallis tests (if 3 arms). We used Spearman rank correlation coefficients (*r*_*s*_) to examine the relation between self-monitoring engagement and change in weight. We also assessed for contamination by exploring whether participants self-monitored when they were not expected to do so.

## Results

### Participant Enrollment and Retention

**Figure 1:**
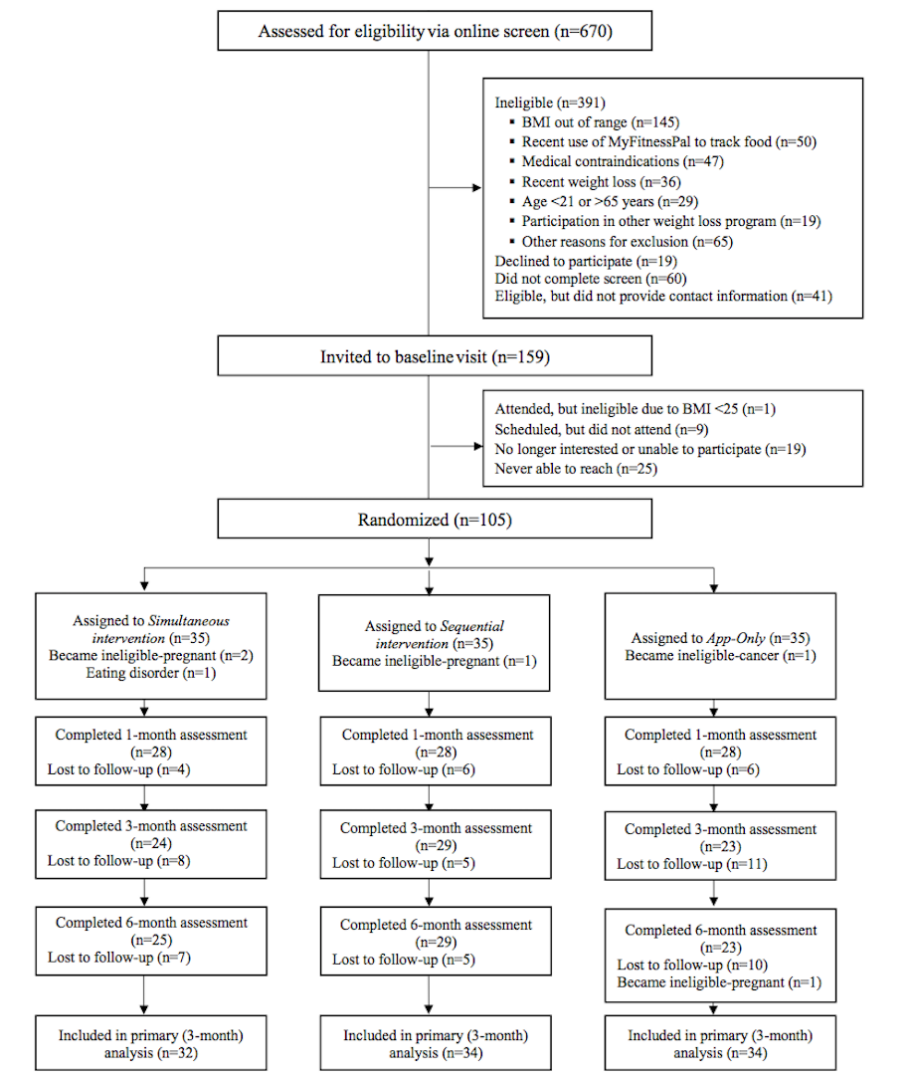
Consolidated Standards of Reporting Trials (CONSORT) flow diagram. BMI: body mass index.

Of the 670 individuals who completed the online screen for eligibility, 58.3% (391) were ineligible, whereas 23.7% (159) were invited to attend the baseline visit. We enrolled 105 participants and randomized them equally to 3 treatment arms (n=35, for each; see Figure 1 for Consolidated Standards of Reporting Trials [CONSORT] diagram). During the trial, 5 participants became ineligible (3 because of pregnancy, 1 because of cancer diagnosis, 1 because of previously undisclosed eating disorder). Of the remaining 100 participants, 84.0% (84 of 100) completed the 1-month visit and 76.0% (76 of 100) completed the 3-month visit. Between 3 and 6 months, one additional participant became ineligible because of pregnancy. At 6 months, 78% (77 of 99) of participants self-reported their weight. Participant retention did not differ significantly between arms at any time point (1 month: *P*=.84; 3 months: *P*=.23; 6 months: *P*=.32). We had no missing self-monitoring engagement data.

### Baseline Characteristics

Table 4 illustrates the baseline characteristics of GoalTracker participants. At baseline, participants had a mean age (SD) of 42.7 (11.7) years and BMI of 31.9 (4.5) kg/m^2^ and were predominantly female (84/100) and employed (78/100). One-third (33/100) were racial or ethnic minorities, most were married or living with a partner (64/100), and the majority had at least a college education (83/100). The majority (56/100) did not track diet in the month before baseline, although most had experience tracking body weight (87.0%). Completers at 3 months differed from noncompleters in race or ethnic minority status (*P*=.03), with 16% (11/67) of non-Hispanic white participants and 39% (13/33) of racial or ethnic minority participants missing the visit.

### Weight Loss

**Figure 2:**
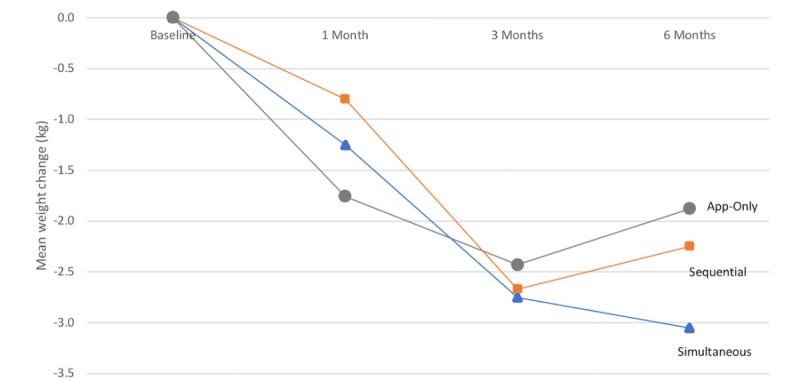
Weight change over time by treatment arm. Data were included for 100 participants; mean (SD) values were estimated using an intention-to-treat analysis with a linear mixed-model.

Figure 2 displays weight change over time by treatment arm. Weight change was significant over time for all arms (see Table 5). In our primary analysis, the Sequential arm did not significantly differ from the App-Only arm in weight change at 1 month (*P*=.06), 3 months (*P*=.78), or 6 months (*P*=.72). In exploratory analyses, the Sequential arm did not differ from the Simultaneous arm in weight change at 1 month (*P*=.36), 3 months (*P*=.92), or 6 months (*P*=.45).

The proportion of participants achieving at least 3% weight loss at 3 months was similar between arms (Sequential: 44%, 15/34 vs App-Only: 29%, 10/34, *P*=.21; exploratory analysis: Sequential vs Simultaneous 41%, 13/32, *P*=.77). Likewise, weight loss of at least 5% at 3 months occurred in 21% of Sequential participants (7/34) and 15% of App-Only participants (5/34), which was not significantly different (*P*=.52). In exploratory analyses, the proportion of participants with ≥5% weight loss did not significantly differ between the Sequential arm and the Simultaneous arm (31% of participants, 10/32, *P*=.32).

### Intervention Engagement

As expected, the Sequential arm tracked *weight* significantly more days than the App-Only arm (who was not asked to track weight) over the 12-week intervention; median (IQR) 70%, (28%-90%) vs 1% (0%-8%), respectively (*P*<.001). In exploratory analyses, the frequency of days participants self-monitored *weight* did not differ between the Simultaneous arm (73%; 25%-90%) and the Sequential arm over 12 weeks (*P*=.92).

**Figure 3:**
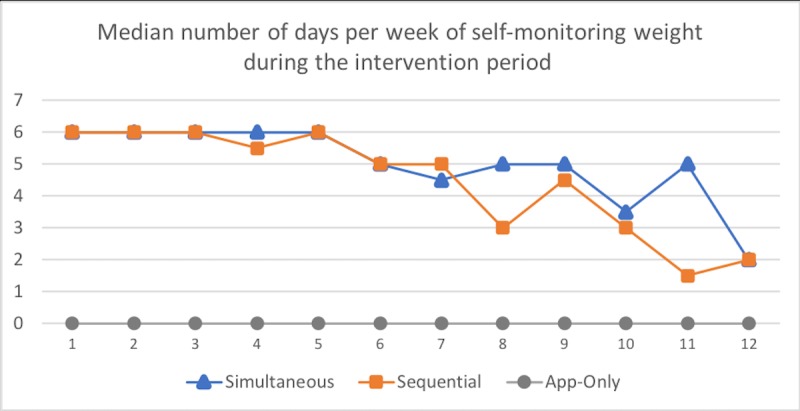
Self-monitoring of weight per intervention week by treatment arm.

**Figure 4:**
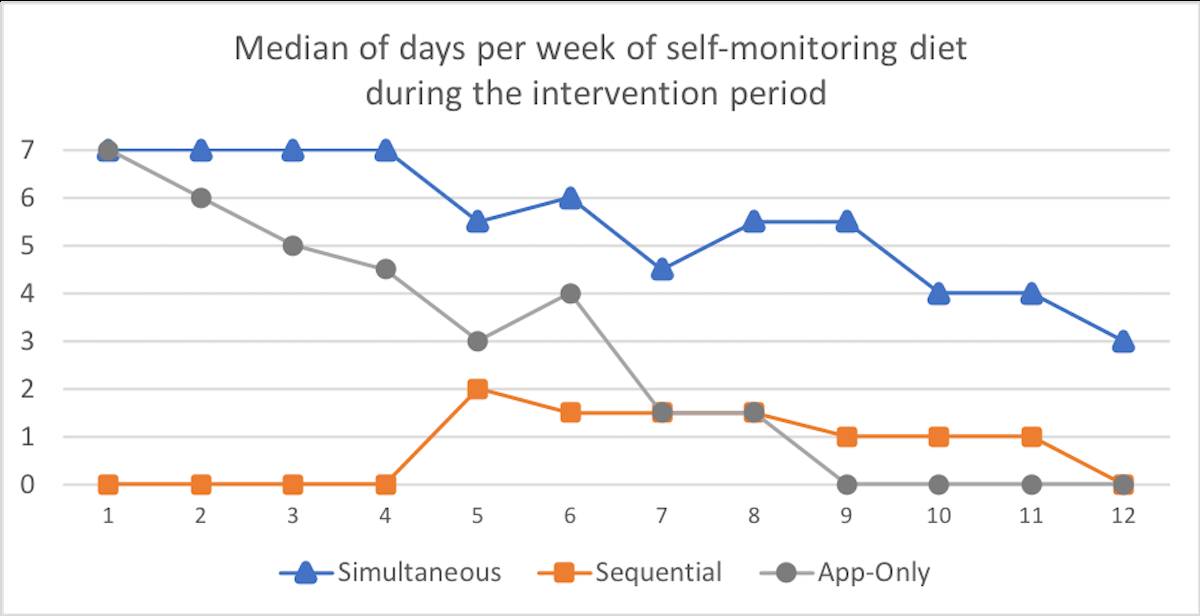
Self-monitoring of dietary intake per intervention week by treatment arm.

As expected, the frequency with which the Sequential arm and the App-Only arm tracked *diet* in weeks 1 to 4 was significantly different (see Table 6). Between weeks 5 and 12 (once the Sequential arm began tracking diet), there was no longer a significant difference between the Sequential and App-Only arms; 27% (4%-80%) vs 21% (0%-62%), respectively (*P*=.54). In exploratory analyses, there were no significant differences in the frequency of days of self-monitoring *diet* between the Simultaneous and App-Only arms over the intervention period; 77% (27%-96%) vs 42% (17%-75%), *P*=.10. Table 6 displays additional self-monitoring and action plan completion outcomes (see Figures 3 and 4 for weekly data).

#### Relation Between Self-Monitoring Frequency and Weight Change

The percentage of days weight was tracked was significantly associated with 3-month weight change in both the Simultaneous arm (*r*_*s*_=−.48, *P*=.02) and the Sequential arm (*r*_*s*_=−.47, *P*=.01). In the same time period, the association between weight change and the percentage of days with complete diet entries was significant in the App-Only arm (*r*_*s*_=−.58, *P*=.003) but not for the Simultaneous arm (*r*_*s*_=−.25, *P*=.24). The percentage of days diet was tracked starting in week 5 for the Sequential arm was significantly associated with weight change at 3 months (*r*_*s*_=−.44, *P*=.02; see Table 7 for additional details).

#### Contamination

The median (IQR) frequency of days that App-Only participants tracked weight in the MyFitnessPal app during the 3-month intervention was 1% (0%-8%), and the frequency of days that Sequential participants tracked diet during month 1 was 0% (0%-0%; see Table 6 for absolute values).

#### Action Plan Completion

In the Simultaneous arm, the median (IQR) number of action plans completed was 7.7 of 11—70% (14%-91%)—compared with 3 of 11—27% (9%-82%)—in the Sequential arm; in exploratory analyses, this difference was not statistically significant (*P*=.21). Percent action plan completion was significantly related to weight change at 3 months in the Sequential arm (*r*_*s*_=−.60, *P<*.001) but not in the Simultaneous group (*r*_*s*_=−.07, *P*=.75).

#### Review of Feedback Email

Most participants (35/52; 67%) reported reading their weekly feedback email at least once per week, whereas 12% (6/52) of participants reported never reading them. In exploratory analyses, there were no significant differences between the Simultaneous and the Sequential arm (*P*=.90).

**Table 4:** Baseline characteristics by treatment arm.

Characteristic at baseline		Total (N=100^a^)	Simultaneous (n=32)	Sequential (n=34)	App-Only (n=34)
Age (years), mean (SD)		42.7 (11.7)	43.8 (13)	42.1 (11)	42.3 (12)
**Gender, n (%)**					
	Male	16 (16.0)	8 (25)	4 (12)	4 (12)
	Female	84 (84.0)	24 (75)	30 (88)	30 (88)
**Marital status, n (%)**					
	Married or living with partner	64 (64.0)	22 (69)	18 (53)	24 (71)
	Not married or living with partner	36 (36.0)	10 (31)	16 (47)	10 (29)
**Race/ethnicity, n (%)**					
	Non-Hispanic white	67 (67.0)	21 (66)	23 (68)	23 (68)
	Non-Hispanic black	22 (22.0)	9 (28)	6 (18)	7 (21)
	Hispanic (all races)	3 (3.0)	0 (0)	2 (6)	1 (3)
	Non-Hispanic other	8 (8.0)	2 (6)	3 (9)	3 (9)
**Education, n (%)**					
	Less than college graduate	17 (17.0)	7 (22)	6 (18)	4 (12)
	College graduate or above	83 (83.0)	25 (78)	28 (82)	30 (88)
**Employment status, n (%)**					
	Employed, full-time	67 (67.0)	20 (63)	27 (79)	20 (59)
	Employed, part-time	11 (11.0)	3 (9)	1 (3)	7 (21)
	Not employed	22 (22.0)	9 (28)	6 (18)	7 (21)
**Annual household income, in US dollars, n (%)**					
	$0-$49,999	26 (26.0)	8 (25)	9 (27)	9 (27)
	$50,000-$99,999	36 (36.0)	14 (44)	12 (35)	10 (29)
	$100,000 or greater	34 (34.0)	9 (28)	11 (32)	14 (41)
	Unknown/not reported	4 (4.0)	1 (3)	2 (6)	1 (3)
Weight, mean (SD), kg		89.6 (16.0)	89.3 (17)	90.8 (17)	88.6 (15)
Body mass index, mean (SD), kg/m^2^		31.9 (4.5)	31.3 (4)	32.6 (5)	31.7 (4)
**Body mass index category, n (%)**					
	Overweight, 25-29.9 kg/m^2^	40 (40.0)	13 (41)	12 (35)	15 (44)
	Class 1 obesity, 30-34.9 kg/m^2^	38 (38.0)	14 (44)	13 (38)	11 (32)
	Class 2 obesity, 35-39.9 kg/m^2^	17 (17.0)	4 (13)	6 (18)	7 (21)
	Class 3 obesity, 40+ kg/m^2^	5 (5.0)	1 (3)	3 (99)	1 (3)
**Self-monitoring of diet frequency, n (%)**					
	Daily	6 (6.0)	1 (3)	2 (6)	3 (9)
	1 to 6 times per week	14 (14.0)	6 (19)	4 (12)	4 (12)
	Less than 1 time per week	24 (24.0)	4 (13)	11 (32)	9 (27)
	Never	56 (56.0)	21 (66)	17 (50)	18 (53)
**Self-monitoring of weight frequency, n (%)**					
	Daily	11 (11.0)	6 (19)	2 (6)	3 (9)
	1 to 6 times per week	35 (35.0)	6 (19)	14 (41)	15 (44)
	Less than 1 time per week	41 (41.0)	13 (41)	16 (47)	12 (35)
	Never	13 (13.0)	7 (22)	2 (6)	4 (12)
**Type of smartphone, n (%)**					
	iPhone	54 (54.0)	17 (53)	16 (47)	21 (62)
	Android	46 (46.0)	15 (47)	18 (53)	13 (38)
MyFitnessPal already on phone before study, n (%)		20 (20.0)	6 (19)	9 (27)	5 (15)

^a^Five participants omitted because they became ineligible during the intervention period.

**Table 5:** Change in weight and body mass index (intent-to-treat).

Outcome by time point		Mean (95% CI)			
		Simultaneous	Sequential	App-Only	Between-group difference (Seq^a^ vs App-Only)^b^
**Weight change from baseline (kg)**					
	1 month	−1.25 (−1.97 to −0.53)	−0.80 (−1.49 to −0.10)	−1.76 (−2.48 to −1.05)	0.97 (−0.03 to 1.97)
	3 months	−2.75 (−4.01 to −1.49)	−2.67 (−3.85 to −1.49)	−2.43 (−3.69 to −1.16)	−0.24 (−1.97 to 1.49)
	6 months^c^	−3.05 (−4.57 to −1.52)	−2.25 (−3.66 to −0.85)	−1.88 (−3.41 to −0.34)	−0.38 (−2.46 to 1.71)
**BMI^d^ change from baseline (kg/m^2^)**					
	1 month	−0.46 (−0.71 to −0.21)	−0.29 (−0.53 to −0.05)	−0.63 (−0.88 to −0.38)	0.35 (0 to 0.69)
	3 months	−0.99 (−1.44 to −0.55)	−0.95 (−1.37 to −0.54)	−0.88 (−1.32 to −0.43)	−0.08 (−0.69 to 0.53)
	6 months^c^	−1.06 (−1.60 to −0.52)	−0.81 (−1.31 to −0.32)	−0.67 (−1.21 to −0.13)	−0.15 (−0.88 to 0.59)

^a^Seq: Sequential self-monitoring intervention arm.

^b^This is the primary comparison; the App-Only arm is the reference group.

^c^One additional participant was omitted in analyses (App-Only arm) at 6-months due to becoming ineligible after the intervention period and before 6-months.

^d^BMI: body mass index.

**Table 6:** Self-monitoring engagement by treatment arm.

Self-monitoring engagement by time period		Median (interquartile range)			
		Simultaneous (n=32)	Sequential (n=34)	App-Only (n=34)	*P* value
**Baseline to 4 weeks (out of 28 days)**					
	Number of days per week tracked weight	6.25 (2.63 to 6.75)	5.75 (3.50 to 6.50)	0 (0 to 0.75)	—^a^
	Number of days per week tracked diet	6.50 (3.88 to 7.00)	0 (0)	5.38 (2.25 to 7.00)	—
	Percentage of days tracked weight	89 (37 to 96)	82 (50 to 93)	0 (0 to 11)	.52^b^; <.001^c^
	Percentage of days tracked diet	93 (55 to 100)	0 (0)	77 (32 to 100)	.37^d^; <.001^c^
**5 to 12 weeks (out of 55 days)**					
	Number of days per week tracked weight	4.50 (0.69 to 6.13)	4.06 (0.75 to 6.63)	0 (0 to 0.25)	—
	Number of days per week tracked diet	4.88 (0.44 to 6.56)	1.88 (0.25 to 5.50)	1.44 (0 to 4.25)	—
	Percentage of days tracked weight	65 (10 to 89)	59 (11 to 95)	0 (0 to 4)	.95^b^; <.001^c^
	Percentage of days tracked diet	70 (6 to 95)	27 (4 to 80)	21 (0 to 62)	.54^c^; .17^e^
**Entire intervention (out of 83 days)**					
	Number of days per week tracked weight	5.08 (1.75 to 6.25)	4.83 (1.92 to 6.25)	0.08 (0 to 0.58)	—
	Number of days per week tracked diet	5.33 (1.83 to 6.67)	—^f^	2.92 (1.17 to 5.17)	—
	Percentage of days tracked weight	73 (25 to 90)	70 (28 to 90)	1 (0 to 8)	.92^b^; <.001^c^
	Percentage of days tracked diet	77 (27 to 96)	—^f^	42 (17 to 75)	.10^d^
	Percentage of action plans completed	70 (14 to 91)	27 (9 to 82)	—	.21^b^
**13 weeks to 6 months (postintervention; out of 99 days)^g^**					
	Number of days per week tracked weight	0.32 (0 to 0.89)	0.43 (0 to 1.64)	0 (0 to 0.14)	—
	Number of days per week tracked diet	0.14 (0 to 1.18)	0 (0 to 0.29)	0 (0 to 0.43)	—
	Percentage of days tracked weight	5 (0 to 14)	7 (0 to 23)	0 (0 to 2)	.78^b^; .004^c^
	Percentage of days tracked diet	3 (0 to 17)	0 (0 to 5)	0 (0 to 6)	.96^c^; .43^e^

^a^Not applicable.

^b^Simultaneous arm versus Sequential arm.

^c^Sequential arm versus App-Only arm.

^d^Simultaneous arm versus App-Only arm.

^e^All arms.

^f^As the Sequential arm did not track diet in the first 4 weeks of the intervention, their results for the “Entire intervention” section would be the same as the results in the “5 to 12 weeks” section above.

^g^One additional participant was omitted in analyses (App-Only arm) at 6 months because of becoming ineligible after the intervention period and before 6-months.

**Table 7:** Spearman rank correlation between engagement metrics and weight change. The table displays correlations and *P* values for the treatment arms that were asked to track during the given time period.

Engagement metric		Weight change by 1 month	Weight change by 3 months
Percentage of action plans completed		—^a^	Both: −.41^b^
		—	Sim^c^: −.07
		—	Seq^d^: −.60^b^
**Baseline to 4 weeks**			
	Percentage of days tracked weight	Both: −.35^b^	Both: −.40^b^
		Sim: −.40^e^	Sim: −.51^e^
		Seq: −.29	Seq: −.34
	Percentage of days tracked diet	Both: −.42^b^	Both: −.40^b^
		Sim: −.36	Sim: −.30
		App^f^: −.51^b^	App: −.48^b^
**5 to 12 weeks**			
	Percentage of days tracked weight	Both: −.44^b^	Both: −.48^b^
		Sim: −.37	Sim: −.49^e^
		Seq: −.54^b^	Seq: −.46^e^
	Percentage of days tracked diet	All^g^: −.37^b^	All: −.42^b^
		Sim: −.24	Sim: −.27
		Seq: −.50^b^	Seq: −.44^e^
		App: −.52^b^	App: −.55^b^
**Entire intervention**			
	Percentage of days tracked weight	Both: −.44^b^	Both: −.47^b^
		Sim: −.40^e^	Sim: −.48^e^
		Seq: −.50^b^	Seq: −.47^e^
	Percentage of days tracked diet^h^	Both: −.35^b^	Both: −.42^b^
		Sim: −.30	Sim: −.25
		App: −.52^b^	App: −.58^b^
**13 weeks to 6 months (post intervention period)^i^**			
	Percentage of days tracked weight	Both: −.50^b^	Both: −.43^b^
		Sim: −.49^b^	Sim: −.43^e^
		Seq: −.59^b^	Seq: −.43^e^
	Percentage of days tracked diet	Both: −.29^b^	All: −.35^b^
		Sim: −.27	Sim: −.17
		Seq: −.42^e^	Seq: −.47^e^
		App: −.20	App: −.39

^a^Not applicable.

^b^*P*<.01.

^c^Sim: Simultaneous self-monitoring arm.

^d^Seq: Sequential self-monitoring arm.

^e^*P*<.05.

^f^App: App-Only self-monitoring arm.

^g^All: All 3 treatment arms.

^h^As the Sequential arm did not track diet in the first 4 weeks of the intervention, their results for the “Entire intervention” section would be the same as the results in the “5 to 12 weeks” section above.

^i^One additional participant was omitted in analyses (App-Only arm) at 6 months because of becoming ineligible after the intervention period and before 6-months.

## Discussion

### Principal Findings

A low-intensity intervention utilizing a commercial app for self-monitoring resulted in comparable weight loss at 3 months, with no variability between the Sequential arm and the “off-the-shelf” App-Only arm. Nevertheless, loss of 3% to 5% of initial weight has been linked to improved health outcomes [46,47], suggesting that GoalTracker is an efficacious intervention for clinically meaningful weight loss.

The addition of evidence-based features such as weekly action plans, behavioral lessons, and tailored feedback did not substantially impact outcomes over and above the core intervention (ie, self-monitoring and in-app feedback) during our 12-week treatment, which parallels findings from several digital health weight loss trials [30,48] but not others [6]. Although most commercial weight loss apps do not include many evidence-based features [49], we suspect that weight loss might still occur with the inclusion of goals, daily self-monitoring, and a daily reminder to track. However, we suspect that had the trial been of a longer duration, the benefit of these enhanced features (eg, weekly lessons) would have become apparent in the findings; indeed, by 6 months, trends suggest continued weight loss at 6 months for the Simultaneous arm and relatively less weight regain in the Sequential arm compared with the App-Only arm.

Given that the GoalTracker trial compared 3 multicomponent interventions, we are unable to isolate the effect of self-monitoring diet and weight, along with each of the additional intervention components. Using a factorial design in consort with the multiphase optimization strategy (MOST) [50] would allow researchers to investigate the unique impact of each intervention component and then build and test an optimized intervention.

We found that self-monitoring engagement was high and that greater frequency of self-monitoring was related to greater weight loss. Contrary to our hypothesis, the Sequential arm did not demonstrate significantly greater engagement in self-monitoring dietary intake than the App-Only arm. In fact, once the Sequential arm was instructed to begin tracking diet after the first month, they tracked only 27% of days. It is possible that the Sequential arm participants’ minimal weight loss in the first month may have negatively impacted their future engagement or that being asked to track diet after a 1-month period seemed like an additional burden that many were unwilling to begin (indeed, almost half or 47% of Sequential participants never or rarely [<20% of days] tracked diet in weeks 5 to 12 [data not shown]). Although the trial was not powered to detect differences between the Simultaneous and Sequential arms, it appears that the Simultaneous arm outperforms the Sequential arm in frequency of tracking diet. This finding suggests that concurrently tracking diet and weight may have reinforced use of the app and activated the overarching goal of losing weight [51], thus leading to high engagement in both entities.

Future studies could consider framing the initial period before self-monitoring diet as a time during which to build self-regulatory skills rather than focus on weight loss, as has been done previously [9]. We selected weight tracking as the precursor to diet tracking for the Sequential arm because we wanted a behavior that could target the theoretical constructs of mastery—considered the best way to strengthen self-efficacy—and self-regulation. Self-weighing is a well-accepted strategy [52] in which mastery can be achieved [16], and self-regulatory capacity can be strengthened [53]. It is possible that providing more rationale for using a sequential approach would have encouraged participants to engage in diet tracking once asked to do so.

### Comparison With Prior Work

Notably, GoalTracker is the first weight loss trial to compare a sequential self-monitoring approach with a traditional approach that asks participants to track multiple components simultaneously. Previous work has compared a simultaneous approach with either a sequential or single component approach in other contexts, with mixed results [21,54]; no examination has focused on self-monitoring or digital approaches for weight loss.

The App-Only arm in GoalTracker performed better than expected. Intervention participants in Laing et al’s trial used the same MyFitnessPal app for diet tracking and lost 0.27 kg at 3 months and 0.03 kg at 6 months and had poor intervention engagement [44]. Possible explanations for the difference in weight loss between Laing et al’s trial and GoalTracker include GoalTracker’s use of specific goals to track diet daily and to lose 5% of initial weight by a specified end date and usage of phone-based reminder notifications.

In comparison with other randomized trials of *commercial* [28,29,32,55-58] or *researcher-* designed [24,59,60] apps for self-monitoring of diet, GoalTracker’s Simultaneous arm tended to have greater adherence to diet tracking, whereas the Sequential arm had lower adherence. Given that most weight loss trials of commercial apps are pilot studies and/or were not powered to detect an effect in weight change between treatment arms [26,28,29,38,56,61], more fully powered studies are needed that examine the efficacy of commercial apps for weight loss.

GoalTracker’s Simultaneous arm had a comparable or higher proportion of participants achieving 5% weight loss compared with other weight loss interventions that used mobile apps for self-monitoring dietary intake (range: 26%-35%) [28,59,62,63] but lower rates than some interventions including counseling (range: 42%-44%) [29,60,61].

### Strengths

Strengths of this trial include the collection of objective self-monitoring data for all participants via an API, use of a popular commercially available smartphone app, and ability to isolate the effect of a sequential versus simultaneous self-monitoring approach. In addition, this trial mimicked real-world weight loss experience (ie, no run-in period, prebaseline visit, or orientation session); consequently, it is possible that removal of these treatment barriers allowed for inclusion of participants with lower motivation and readiness to change. This design may have greater external validity but may make it harder to detect an effect between arms. Another strength was that the trial had little contamination between arms, which has been a problem in past app-based trials where up to 50% of participants in no-treatment control arms were found to have used commercial apps during the study period [44,55,63].

### Limitations

As this study was powered on superiority rather than equivalency, we cannot definitively assert that the treatment arms produce comparable weight loss. In addition, we collected self-reported weight at 6-months because of logistical reasons; however, we were encouraged to find that no additional attrition occurred between 3 and 6 months, despite no contact occurring during that period and no incentive given to provide a weight value. As is common in behavioral interventions, we provided minimal financial compensation to offset costs of attending study visits. Although we acknowledge that financial compensation can serve as an incentive for some to participate—and thus, may result in response bias on self-report measures—we expect this is unlikely, given that compensation was appropriately low. In addition, neither study staff nor participants were blinded to treatment arm, and we required participants to have access to a bathroom scale, although this mimics the real-world population who would track weight. Finally, this study did not include a pure control arm without an intervention, which may have led to an underestimation of treatment effects, as could the possibility of data not actually missing at random.

### Conclusions

This study adds to the limited literature of randomized trials that assess the efficacy of commercially available mobile apps for weight loss [20,26]. In the GoalTracker trial, all 3 versions of the intervention produced weight loss and had high self-monitoring engagement, with no significant impact of additional features nor differential findings between a sequential versus simultaneous approach to self-monitoring. These results suggest that regardless of the order in which diet is tracked, using tailored weight and calorie goals and a commercial app can produce clinically significant weight loss in one-third of individuals. Stand-alone digital health treatments may be a viable option for those looking for a lower intensity approach who are willing and able to track.

## Appendix

Multimedia Appendix 1 Example weekly lesson.

Multimedia Appendix 2 CONSORT‐EHEALTH checklist (V 1.6.1).

## Abbreviations

API: application programming interface
BMI: body mass index
CONSORT: Consolidated Standards of Reporting Trials
IQR: interquartile range
