# Supplementary material for: Comparing Self-Monitoring Strategies for Weight Loss in a Smartphone App: Randomized Controlled Trial
Source: JMIR Mhealth Uhealth. 2019 Feb 28;7(2):e12209. doi: 10.2196/12209 (PMC6416539; doi:10.2196/12209)

10 GoalTracker Lesson 10

EMOTIONAL EATING

It is common for us to eat based on **an emotion** rather than **hunger**.

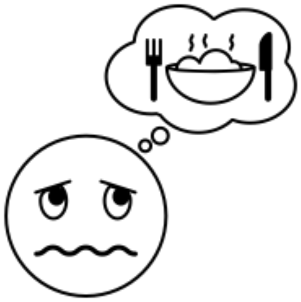

For instance, you may have felt bored, and then wandered into the kitchen. Or perhaps you felt celebratory and ate a huge slice of cake even though you were full. Or maybe, you felt sad and picked up a jar of ice cream.

Many emotions – both negative *AND* positive – can lead us to (1) eat more than we would have done, and (2) eat foods that are high in sugar or fat and low in nutritional quality.

This lesson will focus on recognizing different emotions that may play a role in your eating experience, and brainstorming ways you can make change.

IDENTIFY WHAT EMOTIONS YOU ARE EXPERIENCING

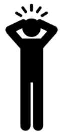

Emotion and Examples

- Feeling Stressed or Overwhelmed – When we engage in mindless eating when feeling like we have a lot going on

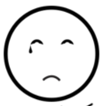

- Feeling Bored – When we turn to our refrigerator when we don't know what else to do
- Feeling Sad or Lonely – When we order an unhealthy takeout when we feel alone

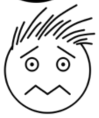

- Feeling Tired – When we turn to soda instead of a nap or a walk outside
- Feeling Tense or Anxious – When we are waiting anxiously for test results and decide to nibble on a bag of chips

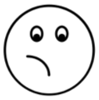

- Feeling Fearful of Failure or Criticism – When we procrastinate on a project, we often turn to food to justify our delay

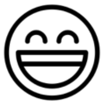

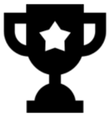

- Feeling Celebratory – When we meet up with a friend whom we haven't seen in a long time and decide to indulge at dinner despite our weight loss goals
- Feeling Motivated – When we motivate ourselves on a task by promising that we can eat food while doing the task or having food as a reward for completion of the task

Remember! Many times we turn to eating when we are trying to improve our mood. The problem with this tendency is two-fold:

1. We tend to eat unhealthy foods when we are eating based on emotions
2. We tend to overeat. Often, our emotions aren't linked to our hunger level, so when we eat in order to try to improve our mood, we find ourselves eating when we actually aren't hungry.

STEPS TO MANAGE EMOTIONAL EATING

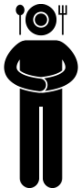

1. **Learn to recognize true hunger**
   a. Not sure? Emotions usually lead to *specific* cravings (think ice cream, brownies, French fries) and an intense desire to eat now. Physical hunger tends to be more gradual

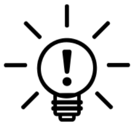

2. **Recognize your emotional triggers** – know which emotions you encounter tend to promote unhealthy eating or overeating

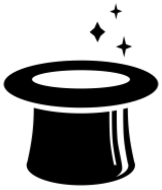

3. **Then…**
   a. **Distract yourself**
   b. **Pick another activity to do**
   c. **or, Accept that you have this emotion.** Let the emotion pass without changing anything else

GENERATE IDEAS TO COPE WITH SUCH EMOTIONS

As mentioned above, when facing emotions that trigger us to eat, it is often helpful to engage in a different activity. Ask yourself these 3 questions to help generate ideas.

Ask yourself:

1. Is this an emotion I want to reduce?
   - *If YES* – Think about a way to cope with the emotion.
   - *If NO* – Think about another way to maintain or enhance this emotion without food.
2. What has worked in the past?

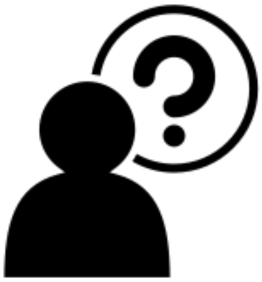

Example activities for different emotions:

- Feeling stressed

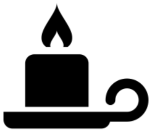

  - Take a brief walk
  - Engage in 10 minutes of deep breathing
  - Make a to do list of a few items that are most important to do now. Hold off on writing down anything else on your list

- Feeling bored

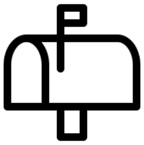

  - Do something productive, such as washing dishes, checking mail, or organizing your closet
  - Set new goals
  - Start a new book
  - Work out while watching a tv show you haven't seen

- Feeling lonely

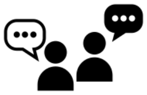

  - Contact a friend to meet up and workout together or do something fun that does not involve food
  - Meet people: take a class, join a team/club, volunteer
  - Start a video chat with a relative you haven't talked to in the past week

- Feeling tired

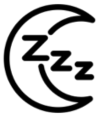

  - Plan your day so that you can get more sleep tonight
  - Take a short (15-20 minute) nap

- Feeling celebratory

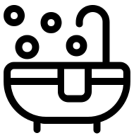

  - Plan something fun to do *now* that does not involve food, such as taking a bubble bath, or playing mini-golf
  - Post about your success on social media or a blog
  - Book a special event for the near future, such as a spa trip, beach outing, or jazz concert

- Feeling motivated

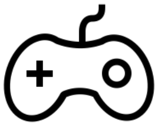

  - When working on a task, play energizing music and take small breaks
  - Set a non-food reward for completing the task, such as playing a video game, watching a movie, or buying an item on your wish list

IMPLEMENT ONE OR MORE OF YOUR IDEAS

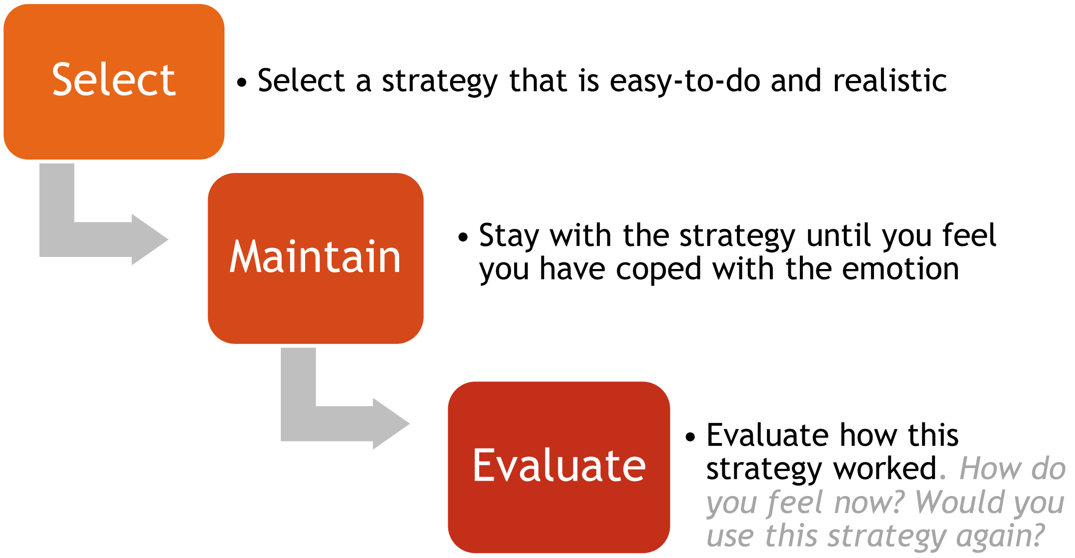

Supplement: Multimedia Appendix 1 [file mhealth_v7i2e12209_app1.pdf]
